# Comparative genomics-based insights into *Pantoea ananatis* strains, isolated from white spot diseased leaves of maize with plant growth-promoting attributes

**DOI:** 10.1128/aem.00329-25

**Published:** 2025-05-19

**Authors:** Fenghuan Yang, Miao Li, Hanxiang Wu, Chao Yu, Wende Liu, Huamin Chen

**Affiliations:** 1 State Key Laboratory for Biology of Plant Diseases and Insect Pests, Institute of Plant Protection, Chinese Academy of Agricultural Sciences 12661, Beijing, China; University of Illinois Urbana-Champaign, Urbana, Illinois, USA

**Keywords:** *Pantoea ananatis*, comparative genomics, evolution, environmental adaptation, maize white spot

## Abstract

*Pantoea ananatis* is a member of the Enterobacteriaceae family known for its broad host adaptability. This study isolated 10 *P*. *ananatis* strains from white spot (MWS)-diseased leaves of maize (*Zea mays*) grown in Yunnan Province, China, and analyzed their putative functions, genomic diversity, and variation. The inoculation tests revealed that none of the 10 isolates caused MWS symptoms in maize. Nine maize isolates, except for S47, induced a hypersensitive response (HR) in tobacco and caused rot symptoms in onion. Most isolates exhibited plant growth-promoting characteristics, with strains JCC14, JCY1, and S47 significantly enhancing maize seedling growth parameters. Genomic sequencing of 10 maize isolates and two rice isolates revealed that 12 isolates clustered into three groups, with an open pan-genome identified. Ancestral reconstruction indicated that the genome size increased in Group A and then decreased in Group B, with significant gains in orthologous groups at Node 14, the most recent common ancestor (MRCA) of Group A and Group B, and at Node 19, the MRCA of seven maize-isolated strains and other Group B strains. Additionally, 11 single-copy orthologous groups were under positive selection. Furthermore, the HIVir (high virulence, also known as PASVIL, *P. ananatis*-specific virulence locus) cluster and type VI secretion system-related genes were conserved in certain *P. ananatis* strains but were not related to their group divergences. This study not only reveals the diverse functions of MWS-diseased maize *P. ananatis* isolates, but also enhances our understanding of divergent genome evolution and environmental adaptation across *P. ananatis* species.

**IMPORTANCE:**

*Pantoea ananatis* is a bacterium commonly found in various agronomic crops. Maize white spot (MWS) has been one of the most destructive diseases affecting maize, leading to significant economic losses. This study clarified that *P. ananatis* strains colonized maize leaves but were not the causal agents of MWS in Yunnan Province, China. Moreover, most of these *P. ananatis* strains exhibited plant growth-promoting (PGP) activities, induced hypersensitive response (HR) activity on tobacco, and caused rot symptoms in onion. Notably, the analysis of divergence throughout the evolutionary process revealed significant genomic evolution and environmental adaptation in these *P. ananatis* strains. This highlights the genetic exchange that has shaped the genome of *P. ananatis*. These findings improve our understanding of the functional diversity of *P. ananatis* strains across different hosts and their positions within the evolutionary lineages of *P. ananatis* species.

## INTRODUCTION

As a Gram-negative bacterium from the Erwiniaceae family, *Pantoea ananatis* exhibits various survival modes, including roles as parasitism, epiphytes, endophytes, and saprophyte, which was initially described as the pathogen responsible for pineapple fruit decay (1). *P. ananatis* has been frequently isolated from various environmental sources, including insects, plants, humans, soil, and water systems (2). This bacterium has demonstrated significant ecological and functional diversity, functioning as both a beneficial plant growth promoter and a pathogen that causes diseases in various crops. Consequently, it is a compelling subject for microbiological studies to provide valuable insights into its dual roles in symbiotic and pathogenic interactions across diverse ecosystems.

It is reported that *P. ananatis* can cause diseases in a variety of crops, including onions, rice, corn, melon, wheat, *Eucalyptus*, and pineapple (2, 3). It can induce certain symptoms such as fruit and bulb rot, dieback, leaf spot, and stalk blight. For instance, onion center rot caused by *P. ananatis* is economically significant, and the bacterium also affects other cultivated *Allium* species, such as leek, shallot, and chive (4–6). In rice, *P. ananatis* can cause a disease with symptoms similar to those of bacterial blight (BB) caused by *Xanthomonas oryzae* pv. *oryzae* (*Xoo*), including spots that spread from the leaf tips, leading to water loss and drying out of the leaves (7–11). Additionally, some non-pathogenic *P. ananatis* strains with plant growth-promoting (PGP) attributes have been identified for their ability to enhance plant growth by producing phytohormones or by improving nutrient uptake through the transformation and transfer of essential nutrients into absorbable forms (12–14). For instance, *P. ananatis* AMG521 isolated from rice paddies produces siderophores, cellulose, and indole-3-acetic acid (IAA) and has the ability to solubilize phosphate, thereby increasing rice yield (15). In addition, certain *P. ananatis* strains exhibit PGP activities and inhibit the growth of various phytopathogens, such as *Xoo*, *Burkholderia glumae*, *Botrytis cinerea*, *Fulvia fulva*, and *Alternaria solani* (16, 17). Therefore, understanding the diverse roles of *P. ananatis* is crucial for developing agricultural management strategies that leverage its beneficial properties while mitigating its pathogenic potential.

The genomic features related to adaptation, resistance, and virulence are typically controlled by dispensable genomes. Comparative genomics based on whole-genome sequencing is an effective method for uncovering bacterial evolutionary and genetic information from pan-genomes. The genome of *P. ananatis* consists of a circular chromosome and a single plasmid (18, 19). The core genomes of *P. ananatis* isolated from various sources and ecological regions exhibit high nucleotide sequence similarity (>99%) (20, 21). Previous comparative genomic analyses have suggested that *P. ananatis* lacks key pathogenicity factors commonly observed in Gram-negative bacteria, such as the type III secretion system (T3SS) and type II secretion system (T2SS) (2, 20). However, multiple virulence factors have been identified, including motility, cell wall-degrading enzymes, quorum-sensing signaling molecules, and effector proteins secreted by the type VI secretion system (T6SS) (22–28).

Maize (*Zea mays* L.) is a crucial crop for food, feed, and industrial raw materials, and plays a significant role in national food security. However, with ongoing global climate change, maize white spot (MWS) has emerged as a severe disease that threatens maize production and causes substantial economic loss (29). *P. ananatis* has been identified as the causal agent of MWS, with reported occurrences in Argentina, Ecuador, Brazil, and China (11, 30–33). In this study, 10 *P*. *ananatis* strains isolated from MWS-diseased maize leaves were examined; however, they did not induce MWS symptoms. We characterized the hypersensitive response (HR) activity of these strains in tobacco and the rot symptoms in onion, and assessed the PGP characteristics of these isolates. Comparative genomic analysis of *P. ananatis* isolates, along with available genomes from public databases, was conducted using phylogeny and pan-genome analyses. Gene content evolution and positive selection were assessed by evaluating the orthologous groups (OGs) of *P. ananatis*. This study aimed to investigate the functions and divergence of *P. ananatis* strains, particularly those isolated from MWS-diseased maize, during evolutionary progression.

## MATERIALS AND METHODS

### Bacterial strains used in this study

Ten *P. ananatis* strains were isolated from MWS-diseased leaves collected in Jiangcheng county, Puer City (N22°46′39.95″, E100°46′39.95″), Yunnan Province, China. Maize leaves were surface-sterilized with 70% ethanol for 1 min, followed by shaking in 1.2% (wt/vol) NaClO solution for 5 min. They were then rinsed three times with sterile distilled water. The leaf tissues were cut into pieces and soaked in sterile ddH_2_O for 5–10 min. The soaked water was spread onto LB plates, which were incubated at 28°C for 24–48 h. Single colonies that grew on the plates were picked and identified by amplifying the 16S rRNA gene using the primer pairs 27f/1492R (34) and the primer PANA_1080 61F/PANA_1080 1009R for *P. ananatis* (35). Additionally, two *P. ananatis* strains isolated from *Xoo*-infected leaves of rice in our previous study were used in this study (17).

### *In planta* evaluation of *P. ananatis* isolates on maize

MWS susceptible maize cultivar (*Zea mays* L.) “Kangnong 2” was used for inoculation assays. *P. ananatis* strains were cultured in LB medium at 28°C until OD_600_ = 0.8. Cells of each strain were resuspended in sterile distilled water. The bacterial suspensions were inoculated on maize leaves at the V3 stage using the leaf clipping method, with distilled water serving as a negative control. Inoculated plants were maintained in a growth chamber at 28°C, 60% relative humidity (11, 22, 30). Simultaneously, the bacterial suspensions were sprayed onto maize plants at the V3 stage grown in a field in Yunnan province, China. Photos were taken on 14 days post-inoculation. Each strain was tested on at least 10 leaves, and all experiments were performed in triplicate.

For bacterial population detection, the plasmid pBBR1MCS-2 or pBBR1MCS-5 was introduced into selected *P. ananatis* strains by electroporation (36). Leaves inoculated with these strains were ground in distilled water using a mortar and pestle, and the mixture was diluted and spread onto LB plates containing Kan (kanamycin) or GM (gentamycin), respectively. The bacterial colonies were counted after incubation at 28°C for 72 h. These experiments were independently repeated three times.

### Pathogenicity of *P. ananatis* isolates on onion

For onions (*Allium cepa* L.) experiments, the outer surface tissue of consumer-grade onions was removed, sterilized in 75% ethanol for 1 min, and promptly rinsed in sterilized water. The onions were wounded using a sterile pipette tip, and 10 µL of prepared bacterial suspension at a concentration with an OD_600_ of 0.8 was inoculated into the wound. The onions were then wrapped with plastic wrap and incubated at 28°C for 4–5 days (27). Symptoms were observed and photographed 6 days post-inoculation.

### HR assay on tobacco

*P. ananatis* strains were grown in LB media for 24 h at 28°C. The cells were then harvested by centrifugation and resuspended in sterilized water at a concentration with an OD_600_ of 0.1. Then the bacterial suspension was infiltrated into the leaves of tobacco (*Nicotiana benthamiana*) using a needleless syringe (37). The symptoms were observed and photographed 24 h after inoculation. Symptoms were observed and photographed 6 days post-inoculation.

### *In vitro* assessment of plant growth-promotion attributes

The PGP traits of *P. ananatis* strains were evaluated using the methods as described previously, including the production of IAA (38), nitrogen fixation (N) (39), solubilization of inorganic phosphate (P) (40), 1-aminocyclopropane-1-carboxylic acid deaminase activity (ACC) (41, 42), lipolytic and proteolytic activity (43, 44), exopolysaccharide (EPS) and cell motility (17, 45). All experiments were repeated at least three times.

### Effects of *P. ananatis* strains on the growth of maize plants

Bacterial strains were cultured in LB medium at 28°C until the OD_600_ reached 0.8. Cells of each strain were resuspended in sterile distilled water. Maize seeds were surface-sterilized with 70% ethanol for 1 min and subsequently washed three times with sterile distilled water. Thirty sterilized seeds were soaked in the bacterial suspension for 1–2 h, with distilled water serving as the negative control. The seeds were then planted in sterilized soil. After 1 month of growth, maize plant height, root length, and wet weight were measured. These experiments were independently repeated three times.

### Inhibition of plant pathogens by *P. ananatis* isolates

The antimicrobial activities of *P. ananatis* strains against three bacterial pathogens (*Xoo*, *Dickeya zeae,* and *Pseudomonas syringae* pv. *actinidiae*) and two fungal pathogens (*Setosphearia turcica* and *Cochliobolus heterostrophus*) were assessed using zone of inhibition tests (17). The bacterial pathogens grown in liquid M210 or LB media were resuspended in sterile distilled water to an OD_600_ of 0.8 and then spread on solid PSA (17) or LB plates using sterilized spreaders. Fungal pathogens were cultured on a certain media plate (30 g/L oat flour, 100 mL/L V8 fruit and vegetable juice, 15 g/L agar). The *P. ananatis* strain isolates were grown on LB plates for 24 h at 28°C. Agar blocks containing *P. ananatis* were placed inversely on the PSA or LB plate with bacterial pathogen and certain media plate with fungal pathogen. Plates inoculated with blank agar blocks served as negative controls. The plates were incubated for 48–72 h at 28°C, and each screening test was repeated three times.

### DNA extraction and sequencing

Bacterial cells of *P. ananatis* were submitted to Beijing Novogene Bioinformatics Technology Co., Ltd. (Beijing, China) for sequencing. Genomic DNA was extracted using the STE method (46). Briefly, bacterial cells were resuspended in STE lysis buffer (0.1 mol/L NaCl, 10 mmol/L Tris-HCl [pH 8.0], and 1 mmol/L EDTA [pH 8.0]). A 10% sodium dodecyl sulfate solution and proteinase K (20 mg/mL) were added and mixed thoroughly. The mixture was then incubated at 56°C for 2 h. DNA was extracted twice using chloroform/isoamyl alcohol (24:1) and water-saturated phenol. DNA precipitation was performed using 3 M sodium acetate (NaAc) and cold anhydrous ethanol at −20°C. After washing twice with 70% ethanol, DNA was dried and dissolved in double-distilled water (ddH₂O). DNA was then quantified with a Qubit fluorometer (Thermo Scientific, USA). A shotgun library with an average insert size of 350 bp was constructed, and the genomes of the *P. ananatis* strains were sequenced on the Illumina PE150 platform using paired-end sequencing. Readfq (version 10) was used to filter the raw data and produce clean data. The raw reads were assembled into contigs using SOAPdenovo (version 2.04) (47, 48), SPAdes (49), and ABySS (50), and the assemblies were integrated using CISA (51). The quality of each genome was assessed with CheckM (52). The gapclose software (version 1.12) was used to optimize the assembly and close gaps, resulting in the final assembly. Fragments below 500 bp were removed, and the contaminated samples were re-decontaminated, followed by evaluation and statistical analysis. GeneMarkS was used to predict and filter genes (53), while coding sequences (CDSs) were annotated through blast searches against NCBI RefSeq non-redundant (NCBI-nr) proteins, Gene Ontology, and Clusters of Orthologous Groups (COG) with an E-value threshold of 1e−5 (54). tRNA, rRNA, and prophage were predicted using tRNAscan SE (Version 1.3.1) (55), rRNAmmer (Version 1.2) (56), and phiSpy (57), respectively. Small RNAs (sRNAs) were first annotated on the Rfam database (58), and final predictions were made using the cmsearch program (version 1.1rc4) (59). CRISPR regions were predicted using CRISPR Digger (Version 1.0) (60). All tools were run with default parameters unless otherwise specified.

### Construction of phylogeny tree and average nucleotide identity analysis

A genome-based phylogeny of *P. ananatis* strains was constructed using the Type Strain Genome Server (TYGS) web server (61). The resulting tree was visualized and refined using the online server iTOL (https://itol.embl.de/) (62). Average nucleotide identity (ANI) values were calculated based on both the BLAST algorithm (ANIb) and the MUMmer algorithm (ANIm), along with the tetranucleotide frequency correlation coefficient (TETRA), via the JSpeciesWS web server (https://jspecies.ribohost.com/jspeciesws/) (63). Heatmaps were generated using the pheatmap function in the pheatmap R package (64).

### Pan-genome analysis

Based on the phylogenetic relationship of *P. ananatis* strains, 22 genomes were chosen for further detailed comparative analysis. The Bacterial Pan-genome Analysis tool (BPGA v1.3) was employed to classify orthologs into core, accessory, and unique genomes. Strains with a higher proportion of unique genes were annotated using the BLAST algorithm against the COG database. Functional classifications of core, accessory, and unique genes were subsequently performed using the BLAST algorithm against the COG and Kyoto Encyclopedia of Genes and Genomes (KEGG) database (65). To estimate the pan-genome and core genome, the USEARCH v11.0.667 program within BPGA was applied, with a 50% cut-off of sequence identity threshold (66). Non-linear fitting was conducted based on the model extrapolation of the pan-genome and core genome.

### Gene content evolution of *P. ananatis* strains

OrthoFinder v2.3.12 was utilized to cluster the protein sequences of *P. ananatis* strains into OGs with default parameters (67). A phylogenetic tree based on single-copy genes was constructed using the EasySpeciesTree v1.0 script with the maximum likelihood (ML) method in RAxML v8.0.26. To investigate the gene content evolution of *P. ananatis*, ancestral gene numbers were inferred using COUNT v9.1106 with Dollo parsimony (68). The gain and loss genes at several key nodes were annotated through the COG database.

### Positive selection analysis

The alignment of single-copy OG sequences was performed using ClustalW v2.1 (69), and the necessary file formatting was completed with ParaAT v1.0 (70). The non-synonymous and synonymous substitution (dN/dS ratio) for each single-copy OG were calculated by the PAML4 codeml program (71). Additionally, the OGs with dN/dS ratio >1 were categorized into COG classifications using blastp.

### HIVir genes and type VI secretion system analysis

According to previous studies of HIVir (high virulence, also known as PASVIL, *P. ananatis*-specific virulence locus) in *P. ananatis* LMG 20103 and T6SS in *P. ananatis* LMG2665 (24, 72), the amino acid sequences of 12 HIVir genes and 19 genes associated with T6SS in 22 *P*. *ananatis* strains were searched and downloaded from the NCBI database. The multiple amino acid sequence alignments of HIVir and the genes associated with T6SS in *P. ananatis* were performed using the ClustalW method in MEGA7 (73). The similarities and differences between the sequences were then analyzed.

## RESULTS

### *P. ananatis strains* colonized maize but did not lead to MWS symptoms

**Fig 1:**
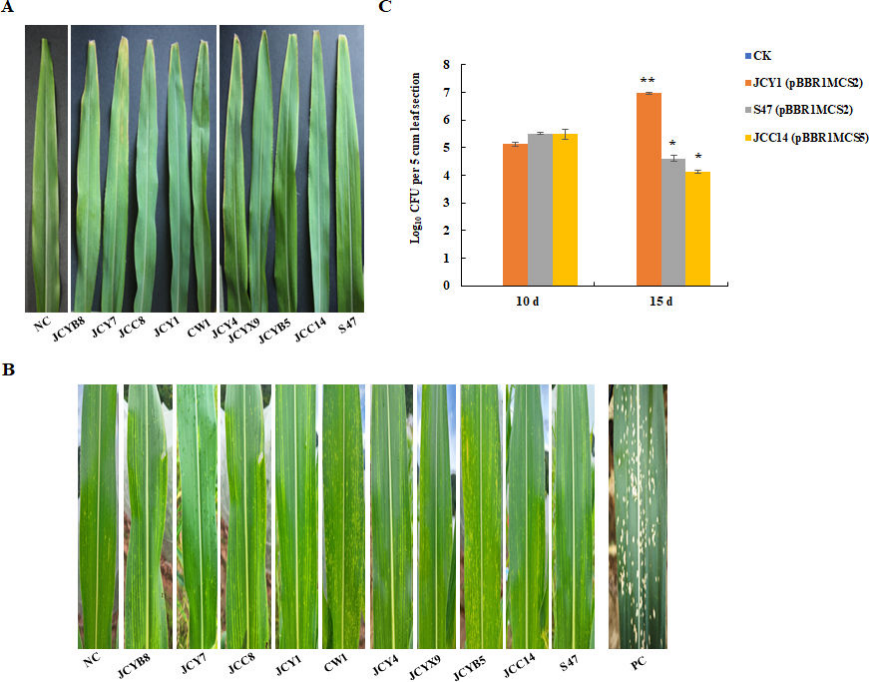
Inoculation assays of *P. ananatis* with maize. Each leaf was inoculated with *P. ananatis* isolates using the leaf clipping method (**A**) and spray method (**B**). Symptoms were observed on the 15th day after inoculation. Meanwhile, colonization of three *P. ananatis* isolates in maize leaves was detected (**C**). NC, negative control; PC, WMS-diseased leaves collected from the field. * indicates *P* < 0.05, as determined by *t*-test.

In this study, 10 *P*. *ananatis* strains were isolated from maize leaves affected by MWS in Yunnan Province. To investigate the role of these isolates in the occurrence of MWS, various inoculation methods were used, including leaf cutting and spraying. The results indicated that none of the 10 strains induced MWS symptoms in maize leaves (Fig. 1A and B). Among these strains, three were further examined for their colonization potential using the leaf-cutting method. As depicted in Fig. 1C, the concentration of JCY1 increased from 10 days to 15 days, whereas that of S47 and JCC14 decreased. These results suggest that *P. ananatis* may not be the causal pathogen of MWS in Yunnan.

### *P. ananatis* strains induce HR in non-host tobacco and cause disease symptoms in onion

**Fig 2:**
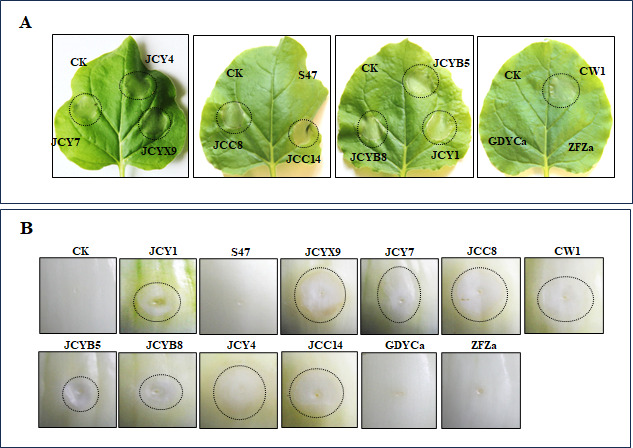
Hypersensitive response assay of *P. ananatis* strains on non-host tobacco and pathogenicity on onion. Bacterial suspensions were infiltrated into tobacco leaves, with at least 10 leaves inoculated per strain in each experiment. Photos were taken 24 h after infiltration into tobacco (**A**). Similarly, bacterial suspensions were infiltrated into onions, and photos were taken 5 days post-infiltration (**B**). The infiltrated regions are marked by black dotted lines. The experiment was repeated three times.

Numerous *P. ananatis* isolates have been reported to elicit HR in tobacco (6, 8, 9, 31). In this study, we first tested HR induction on tobacco using 10 *Pantoea* strains isolated from maize and two strains (GDYCa and ZFZa) previously isolated from rice. The results revealed that nine strains (JCYB5, JCYB8, JCY1, JCY4, JCY7, CW1, JCYX9, JCC8, and JCC14) induced HR at 24 h post-inoculation, whereas strains S47, GDYCa, and ZFZa did not (Fig. 2A). To assess whether these *P. ananatis* strains infect onion, bacterial strains were infiltrated into onion at an OD_600_ of 0.8. The results showed that, similar to the HR induction on tobacco, nine *P. ananatis* strains caused rot symptoms in onion, whereas S47, GDYCa, and ZFZa did not (Fig. 2B). These observations suggested that JCYB5, JCYB8, JCY1, JCY4, JCY7, CW1, JCYX9, JCC8, and JCC14 induced HR in tobacco and caused rot symptoms in onion.

### *P. ananatis* strains exhibited diverse PGP traits

The effects of various *P. ananatis* strains can range from pathogenic to benign or even growth-promoting (21). Certain strains of *P. ananatis* with PGP traits have been identified as potential antagonists of plant pathogens (74). Our previous studies have characterized some strains, such as ZFZa and GDYCa, which can exhibit multiple PGP characteristics and inhibit the growth of *Xoo in vitro*, thereby suppressing BB in rice (17). In this study, we tested 10 newly isolated *P. ananatis* strains for ACC deaminase activity, EPS production, cell motility, phosphate solubilization, secretion of proteolytic enzymes, and antibacterial or antifungal activity. All 10 strains exhibited EPS production, cell motility, ACC deaminase activity, and nitrogen fixation but lacked IAA production and proteolytic enzyme activity (Table 1 and Fig. S1). Additionally, seven strains (JCYB8, S47, JCY4, CW1, JCYX9, JCY7, and JCC8) showed phosphate solubilization, and three strains (JCYB8, JCYB5, and JCY4) demonstrated lipolytic enzyme activity (Table 1 and Fig. S1). Notably, JCY1 inhibited the growth of *Xoo*, which served as the causal agent of bacterial blight in rice (Table 1 and Fig. S2), whereas the other strains did not exhibit inhibitory effects against the selected plant bacterial or fungal pathogens.

**TABLE 1:** Characterization of *in vitro* plant growth-promoting traits of 10 *P. ananatis* strains^*a*^

Strains	IAA	P	N	ACCD	EPS	Cell motility		Secretion of proteolytic enzyme		Antibacterial activity			Antifungal activity	
						Swimming	Swarming	Lipolytic	Proteolytic	*Xoo*	*Psa*	*Dz*	*Setosphearia turcica*	*Cochliobolus heterostrophus*
JCYB8	−	+	+	+	+	+	+	+	−	−	−	−	−	−
JCYB5	−	−	+	+	+	+	+	+	−	−	−	−	−	−
JCY1	−	−	+	+	+	+	+	−	−	+	−	−	−	−
JCC14	−	−	+	+	+	+	+	−	−	−	−	−	−	−
S47	−	+	+	+	+	+	+	−	−	−	−	−	−	−
JCY4	−	+	+	+	+	+	+	+	−	−	−	−	−	−
CW1	−	+	+	+	+	+	+	−	−	−	−	−	−	−
JCYX9	−	+	+	+	+	+	+	−	−	−	−	−	−	−
JCY7	−	+	+	+	+	+	+	−	−	−	−	−	−	−
JCC8	−	+	+	+	+	+	+	−	−	−	−	−	−	−

^*a*^ IAA, indole acetic acid production; P, phosphate solubilization; N, nitrogen fixation; ACCD, ACC deaminase activity; EPS, exopolysaccharide production; swimming and swarming motility, lipolytic and proteolytic activity. *Xoo*, *Xanthomonas oryzae* pv *oryzae* PXO99^A^; *Psa*, *Pseudomonas syringae* pv. *actinidiae *3.2; *Dz*, *Dickeya zeae* EC1. Each experiment was performed with three biological replicates. +, positive detection; −, negative detection.

**Fig 3:**
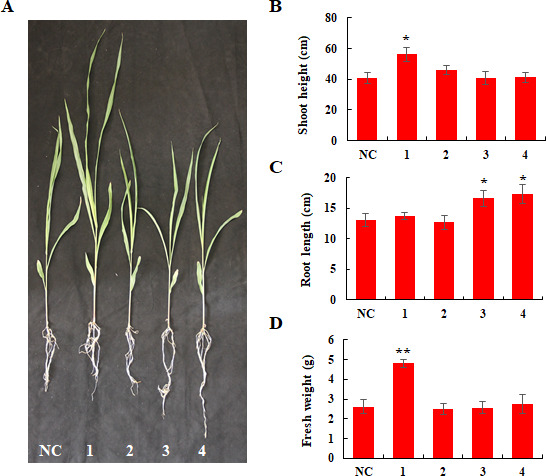
Growth-promoting effects of *P. ananatis* isolates on maize seedlings. Maize seeds were soaked in a bacterial solution at a concentration of 10^8^ cells/mL for 1–2 h, with distilled water serving as a negative control (NC). The seeds were then planted in soil and grew for 1 month. Agronomic traits of maize (**A**) were measured, including shoot height (**B**), root length (**C**), and fresh weight (**D**). Thirty seeds were inoculated with each strain per experiment, and the experiment was repeated three times. * and ** indicate *P* < 0.05 and *P* < 0.01 as determined by *t*-test, respectively. NC, negative control; 1, JCC14; 2, JCYB8; 3, JCY1; 4, S47.

To investigate the effects of *P. ananatis* strains on maize growth, four strains (JCY1, JCC14, JCYB8, and S47) were selected to assess their impact on shoot height, root length, and fresh weight of maize (Fig. 3A). The results showed that strains JCC14 significantly increased shoot height (56.17 ± 4.75 cm) and maize seedling fresh weight (4.81 ± 0.22 g) (*P* < 0.05) compared to the control plants (Fig. 3B and D). Strains JCY1 (16.59 ± 1.26 cm) and S47 (17.27 ± 1.56 cm) significantly enhanced seedling root length (*P* < 0.05) (Fig. 3C). In conclusion, these findings demonstrate that some of these *P. ananatis* strains have a growth-promoting effect on maize seedlings.

### General genomic features of *P. ananatis* strains

The draft genomes of 12 *P*. *ananatis* isolates were sequenced, and their genomic features are summarized in Table 2. Eleven of the 12 genomes were 100% complete with less than 1% contamination, which ensured the reliability of subsequent analyses. The genome lengths ranged from 4,578,911 to 5,124,355 bp, and the CDS ranged between 4,383 and 5,012. The GC content varied from 53.22% to 53.76%. The predicted tRNA counts ranged from 63 to 66, prophages from 1 to 9, and CRISPR sequences from 1 to 5, with no sRNA identified (Table 2).

**TABLE 2:** General features of the newly sequenced *P. ananatis* strains in this study

Strain	Accession no. (SRA database)	Completeness (%)	Contamination (%)	Total bases (bp)	GC (%)	Contigs	CDSs (total)	rRNAs (5S, 16S, 23S)	tRNA	sRNA	Prophage	CRISPR
JCY1	SRR29778427	100	0.08	4,578,911	53.56	26	4,383	7, 2, 1	66	0	4	1
JCY4	SRR29778426	100	0.23	5,071,591	53.44	27	4,992	5, 2, 1	66	0	5	5
JCY7	SRR29778423	100	0.14	4,947,816	53.46	29	4,764	6, 1, 1	63	0	2	5
JCYX9	SRR29778422	100	0.14	4,945,816	53.46	23	4,764	6, 2, 1	66	0	4	3
JCYB5	SRR29778421	100	0.19	4,919,623	53.48	22	4,738	6, 2, 1	63	0	9	3
JCYB8	SRR29778420	100	0.23	5,068,575	53.45	25	4,980	7, 2, 1	63	0	5	0
CW1	SRR29778419	100	0.14	4,947,704	53.46	26	4,767	6, 1, 1	65	0	2	4
S47	SRR29778418	100	0.14	4,813,505	53.76	70	4,762	6, 1, 1	63	0	9	1
JCC8	SRR29778417	100	0.14	4,951,127	53.46	28	4,770	6, 2, 1	64	0	2	3
JCC14	SRR29778416	100	0.14	4,922,173	53.5	24	4,727	6, 3, 1	65	0	1	5
ZFZa	SRR29778425	100	6.33	5,124,355	53.45	21	5,018	6, 1, 1	66	0	4	1
GDYCa	SRR29778424	100	0.08	4,886,978	53.22	32	4,768	7, 1, 1	63	0	5	0

Functional analysis of the isolates revealed that 74.82%–78.19% CDSs of these genomes were annotated and assigned to 24 functional clusters based on COG classification (Table S1).The five most abundant functional categories were G (carbohydrate transport and metabolism, 8.8%–9.46%), E (amino acid transport and metabolism, 8.2%–9.05%), K (transcription, 6.84%–7.48%), M (cell wall/membrane/envelope biogenesis, 5.77%–6.16%), and J (translation, ribosomal structure, and biogenesis, 5.41%–6.11%) across all strains (Table S1). These COG categories of CDSs were involved in bacterial growth, reproduction, and genetic information transmission, which may enhance the adaptability and survival of *P. ananatis* under varying environmental conditions.

### Phylogenetic analysis of *P. ananatis* strains

**Fig 4:**
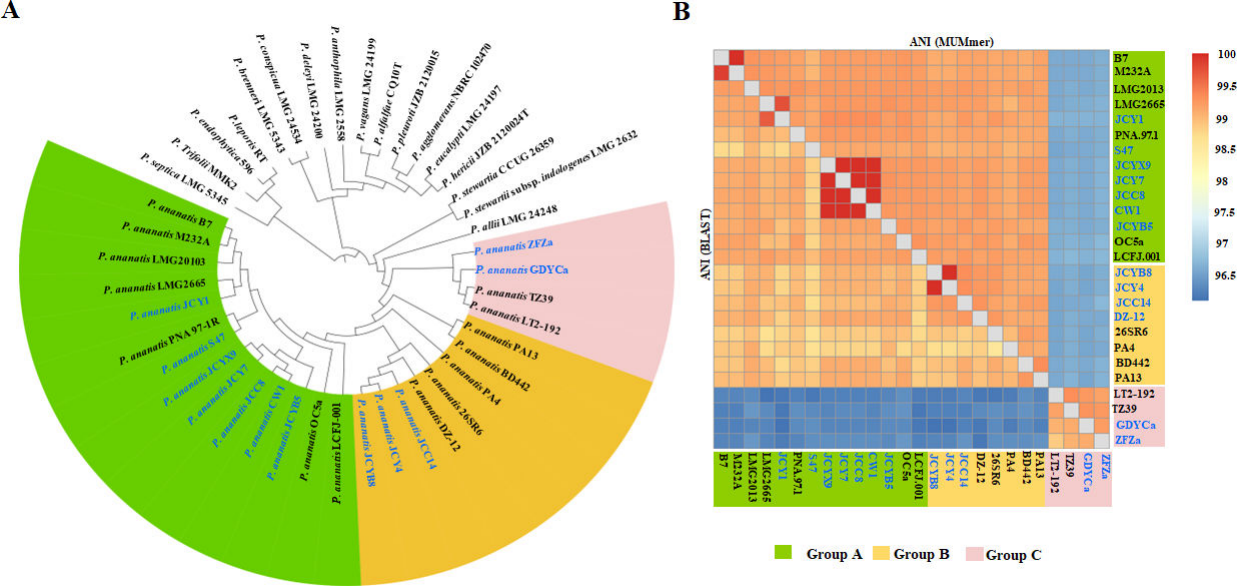
Determination of taxonomic status of 12 newly sequenced *Pantoea ananatis* isolates. (**A**) Phylogenetic tree of 12 sequenced isolates and 14 other *Pantoea ananatis* strains from the GenBank database was constructed using TYGS based on genome BLAST distance phylogeny distances calculated from genome sequences. The complete/draft genome sequences of 10 *Pantoea* sp. strains were automatically deposited using TYGS. Notably, the front of the newly sequenced strains is marked in blue. The strains highlighted in green, orange, and pink represent Group A, Group B, and Group C, respectively. (**B**) Heat map of ANI values using both the blast and MUMmer methods; detailed values are listed in Table S3. The higher the ANI value, the closer the genetic relationships between the strains. The cut-off value proposed for species distinction is approximately 95%–96%.

A phylogenetic tree based on whole-genome sequences was constructed to evaluate the relationships between 12 newly sequenced strains and 14 previously sequenced *P. ananatis* strains (Table S2). The dendrogram indicated that the 12 newly sequenced strains clustered into three groups. Although it is challenging to distinguish *P. ananatis* strains in Group A and Group B, the similarity among *P. ananatis* strains in Group A is higher than that in Group B based on the results of the phylogenetic analysis (Fig. 4A) and ANI values (BLAST) (Fig. 4B). Group A comprised seven new strains (JCY1, S47, JCYX9, JCY7, JCC8, CW1, and JCYB5) and five previously sequenced strains (B7, M232A, LMG2013, LMG2665, and PNA97-1R) (Fig. 4A). Group B included strains JCYB8, JCY4, and JCC14, which were closely related to DZ-12 and 26SR6. Group C contained four strains (ZFZa, GDYCa, TZ39, and LT2-92) isolated from BB-diseased or healthy rice leaves, distinguishing them from the other 22 strains (Fig. 4A). This clustering indicated genomic differentiation among the 26 *P*. *ananatis* strains.

ANI, based on pairwise genome comparisons of shared orthologous genes, can be commonly used for species circumscription in prokaryotes (75). To infer the phylogenetic relationships among the *P. ananatis* strains, ANIb, ANIm, and TETRA values were calculated. As illustrated in Fig. 4B and Table S3, 26 *P*. *ananatis* strains exhibited high ANIb (≥96.1%), ANIm (≥96.5%), and TETRA (≥0.997) values. ANI values ≥95% and TETRA values ≥0.99 were standards for defining organisms of the same species (76). The ANI results confirmed that all the newly sequenced strains belonged to *P. ananatis*, demonstrating high similarity across the 26 strains. Although the four Group C strains exhibited high sequence identity with each other (≥99%) and their ANIb and ANIm values (~96%) relative to the other 22 strains, indicating a relatively lower similarity between Group C and the other strains.

### Core pan-genomic analysis of *P. ananatis* strains

**Fig 5:**
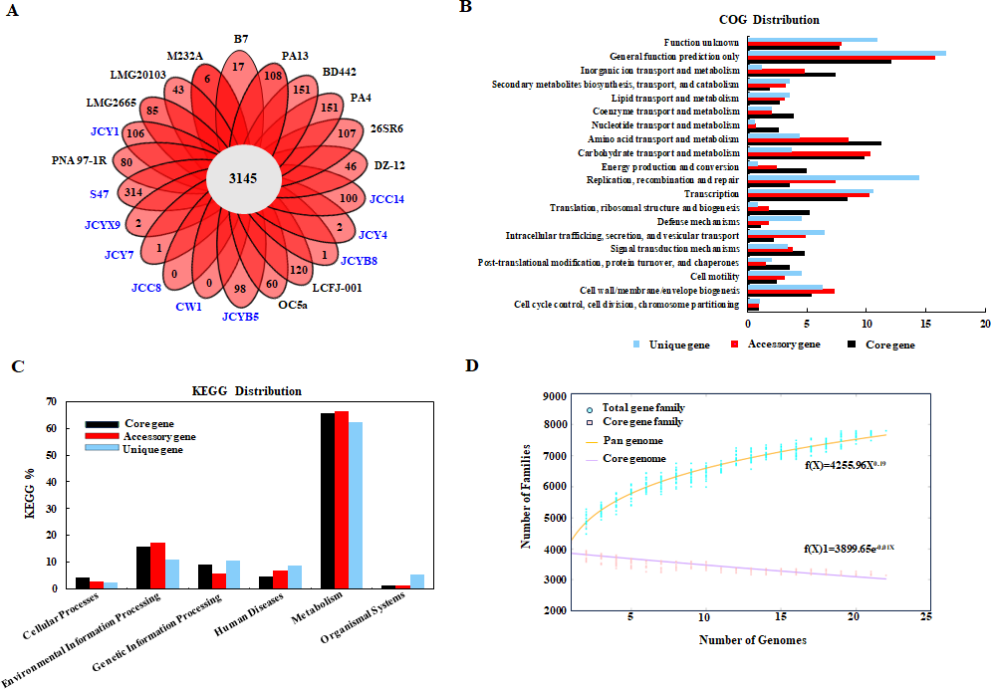
Pan-genome analysis of 22 *P. ananatis* strains. (**A**) Flower plot showing the number of unique genes (in the petals) for each strain and the number of core genes common to all strains (in the center). (**B**) COG classification of core, accessory, and unique genes. (**C**) KEGG pathway assignments of core, accessory, and unique genes. (**D**) Mathematical extrapolation estimating the size of the *P. ananatis* pan-genome and core genome.

To understand the pan-genome of *P. ananatis*, CDSs from the genomes of 22 selected *P. ananatis* strains in Groups A and B were clustered into 6,297 gene families using BPGA. The dendrograms generated through pan-genomic pipelines revealed a core genome consisting of 3,145 protein CDSs, representing 63%–77.4% of each genome in the selected population (Fig. 5A). This percentage was lower than the 69.6%–89.5% core gene coverage observed in eight other *P. ananatis* genomes (3,876) (20). The number of unique genes varied from 1 to 317 across *P. ananatis* genomes. Among the newly sequenced strains, S47 had the highest number of unique genes (317), whereas JCC14, JCYB5, and JCY1 had 100, 98, and 106 unique genes, respectively. Ten selected genomes exhibited more than 30 unique genes, particularly BD442, PA4, and LCFJ-001, suggesting adaptation to different host environments through diverse strategies. In contrast, four new genomes (JCY4, JCYB8, JCYX9, and JCY7) had only 1–2 unique genes, with JCC8 and JCYX9 presenting none, indicating close genetic relationships among these *P. ananatis* strains.

Core, unique, and accessory genes were annotated using COG and KEGG databases. Core genes, typically housekeeping genes, are involved in essential cellular processes and are primarily vertically inherited from the parent to the progeny. The core genomes of 22 *P. ananatis* strains were associated with the transport and metabolism of amino acids (11.2%), carbohydrates (9.7%), inorganic ions (7.3%), transcription (8.3%), and cell wall/membrane/envelope biogenesis (5.3%) (Fig. 5B). These metabolic profiles are crucial for nutrient uptake and maintenance of basic bacterial functions. Most unique genes were related to information storage and processing, including COG categories L (replication, recombination, and repair, 14.2%) and K (transcription, 10.5%), which are essential for environmental adaptation, genomic stability, and survival under harsh conditions (Fig. 5B, Fig. S3, and Table S4). The accessory genes that are prone to lateral gene transfer were primarily associated with ecological niche adaptation and metabolism, including COG categories G (carbohydrate transport and metabolism, 8.4%), L (replication, recombination, and repair, 7.3%), and M (cell wall, membrane, and envelope biogenesis, 7.2%) (Fig. 5B). KEGG classification indicated that metabolism and environmental information processing were the dominant pathways for the core, unique, and accessory genes (Fig. 5C), highlighting the flexible role of these genomes in environmental adaptation.

To determine the expansion of the pan-genome in the 22 *P. ananatis* strains, mathematical extrapolation was conducted using BPGA (Fig. 5D). The pan-genome contained 7,801 gene families and was fitted to an empirical power law regression function using the Allometric1 model [f(X) = 4,255.96X^0.190364^] with a parameter of 0.190364 (Fig. 5D). This parameter, between 0 and 1 and close to 0, indicated that the pan-genome of these 22 *P. ananatis* strains was “open” (77). Furthermore, the number of core genes decreased with increasing number of genomes, fitting an exponential regression using the Exp2PMod1 model [f(X)1 = 3,899.65e^−0.0114928X^]. The curve stabilized at 3,145 core genes when considering 20 genomes (Fig. S4), suggesting that the *P. ananatis* genome size has not yet reached saturation and reflects the species’ diverse habitats and lifestyles.

### Gene content variation

**Fig 6:**
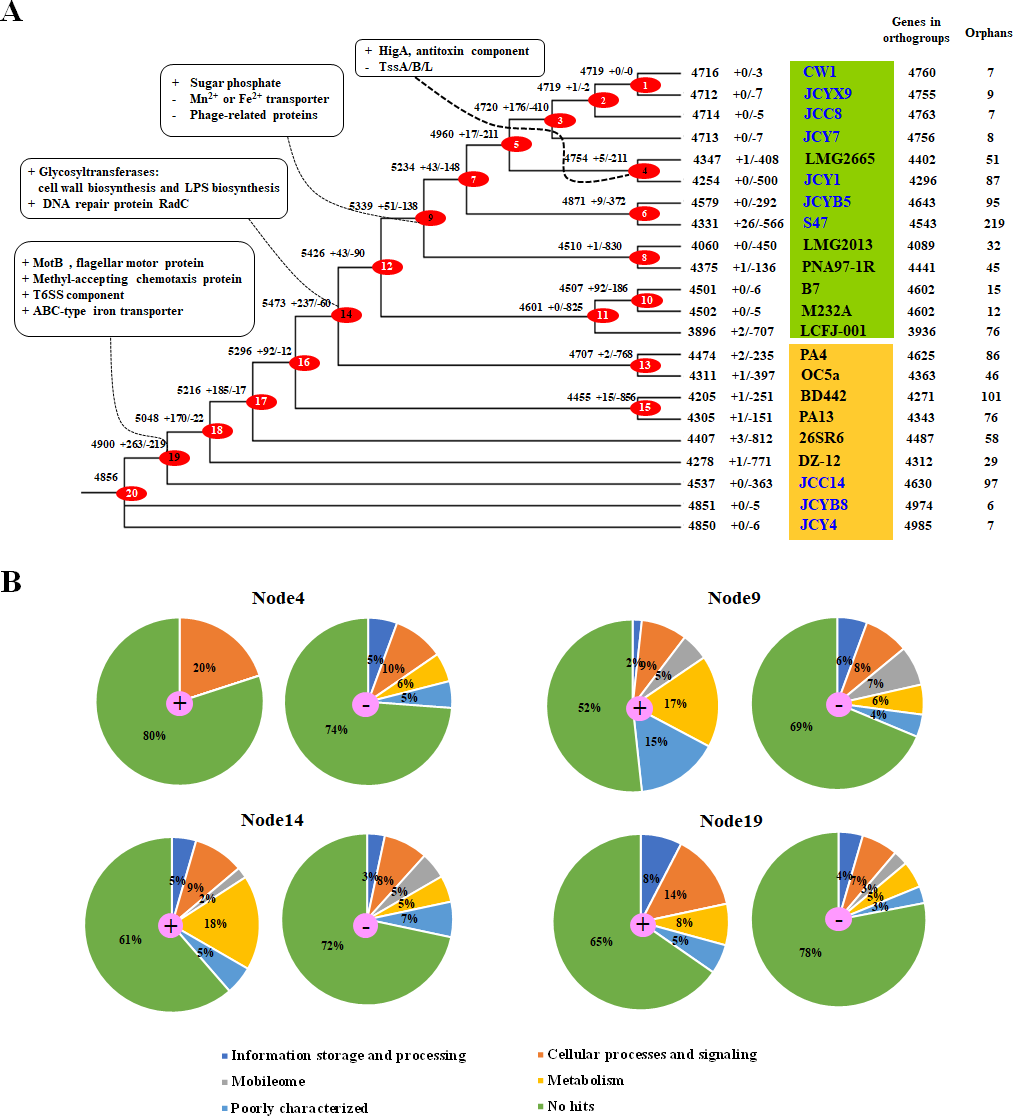
Homologous gene analysis of 22 *P. ananatis* strains using OrthoFinder. (**A**) Phylogenetic tree constructed using the ML method based on the comparison of 3,050 single-copy genes across the 22 *P. ananatis* strains. Ancestral genome reconstruction for the *P. ananatis* genus was performed using Dollo parsimony algorithms implemented in COUNT software. The numbers on the branches represent the gain (+) and loss (−) of genes, respectively, whereas black numbers indicate extant gene counts. (**B**) Pie charts show the COG categories of gain and loss genes at key nodes, with genes involved in gain and loss events labeled in the corresponding clades.

The divergence among *P. ananatis* strains was analyzed by mapping the parsimony-based gene gain and loss events of OGs onto phylogenomic trees constructed from 3,050 single-copy genes (Fig. 6 and Table S5). The analysis revealed an initial increase in the number of OGs within Group A, followed by a significant decrease from groups A to B (Fig. 4A). For most *P. ananatis* strains, phylogenetic relationships were consistent with those based on whole-genome analysis (Fig. 6A), such as those for JCY1, LMG2665, BD442, PA13, B7, and M232A. Gene acquisition and loss are the key mechanisms in bacterial evolution (78), with a substantial number of OGs gained at Node 14, the MRCA of Group A and Group B, and Node 19, the MRCA of new strains (JCC14, JCYB8, and JCY4), and other Group B strains, accounting for 5.3% and 4.3% of OGs, respectively.

Most of the gained and lost genes were related to cellular processes and signaling at Nodes 4 and 19, followed by metabolism and information storage and processing. In addition, numerous genes related to metabolism were identified at Nodes 9 and 14, while many genes associated with cellular processes and signaling were lost at Node 9 and Node 14 (Fig. 6B and Table S6). For example, the antitoxin component HigA was acquired at Node 4, the MRCA of strains LMG2665 and JCY1 (Fig. 6A and Table S6). Node 9 was particularly significant in distinguishing between the seven newly sequenced strains and other selected *P. ananatis* strains in Group A. Genes encoding sugar phosphate were gained at Node 9, while the genes encoding proteins associated with heavy metal transport (Mn^2+^ or Fe^2+^) and seven genes encoding phage-related proteins were lost at this node. The genes encoding glycosyltransferases involved in cell wall biosynthesis and lipopolysaccharide (LPS) biosynthesis, and the DNA repair protein RadC, were obtained from Node14 (Fig. 6A and Table S6). Notably, some genes encoding flagellar rotary motor MotB, methyl-accepting chemotaxis proteins (MCPs), T6SS-related proteins, Mn^2+^ or Fe^2+^ transporters, and ATP-binding cassette (ABC) transporters, FetAB, were acquired at Node 19.

### Positive selection promotes environmental adaptation

In genetics, the dN/dS ratio can be used to evaluate the balance between non-synonymous and synonymous substitutions at a specific site over a given period. Therefore, we calculated dN/dS ratios for single-copy OGs using the PAML4 codeml program. A total of 3,050 single-copy OGs were identified in 22 *P. ananatis* genomes, while 11 of these OGs demonstrated a dN/dS ratio greater than 1, including three OGs annotated as ribosomal proteins (L23, L33, and L35), one OG related to the CsrA/B/C/D system, one OG related to outer membrane murein-binding lipoprotein Lpp, one OG related to DNA-binding winged helix-turn-helix (wHTH) domain, one OG related to a predicted membrane protein encoding in cydAB operon and four unannotated OGs without any predicted functional domain (Table 3). Based on COG classification, the functions of genes under positive selection were associated with categories K (transcription), J (translation, ribosomal structure, and biogenesis), T (signal transduction mechanisms), and M (cell wall/membrane/envelope biogenesis) (Table 3). These results suggest that 11 genes were positively selected in the evolution of the selected 22 *P. ananatis* strains.

**TABLE 3:** Detailed information of 11 single-copy OGs with dN/dS > 1^*a*^

OG number	COG number	COG category	Description
OG0000219	COG0267	J	Ribosomal protein L33
OG0000242	COG0291	J	Ribosomal protein L35
OG0000279	COG1551	T	sRNA-binding carbon storage regulator CsrA
OG0000262	COG4238	M	Outer membrane murein-binding lipoprotein Lpp
OG0000271	COG0089	J	Ribosomal protein L23
OG0002021	COG3790	S	Predicted membrane protein, encoded in cydAB operon
OG0001943	\^*b*^	\	\
OG0000998	\	\	\
OG0002626	\	\	\
OG0002978	\	\	\
OG0002520	COG3710	K	DNA-binding winged helix-turn-helix (wHTH) domain

^*a*^ K, transcription; S, function unknown; J, translation, ribosomal structure, and biogenesis; T, signal transduction mechanisms; M, cell wall/membrane/envelope biogenesis.

^*b*^ \, no hits.

### HIVir gene cluster in *P. ananatis* strains

**Fig 7:**
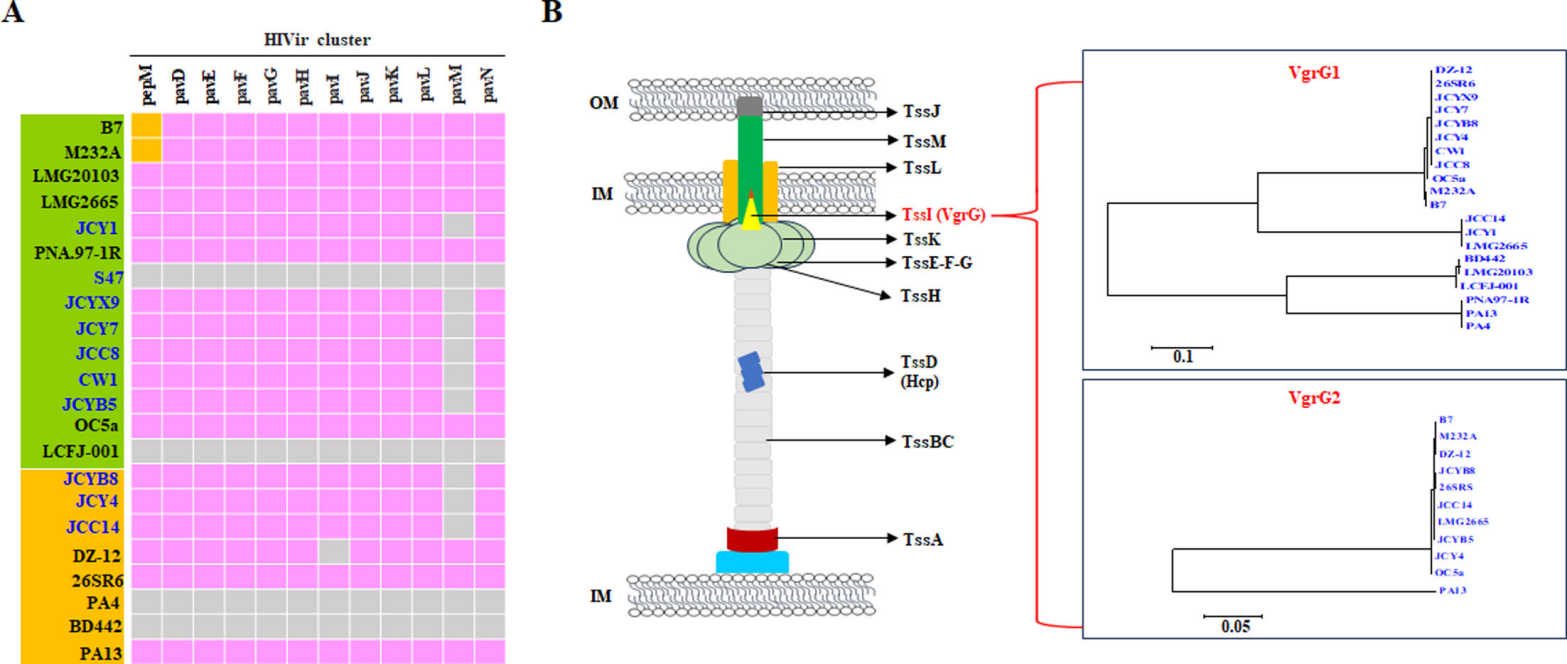
HIVir (**A**) and T6SS-related genes (**B**) in 22 *P. ananatis* strains. (**A**) The presence or absence of 12 gene-related HIVir clusters was analyzed. Strains highlighted in green and orange represent Group A and Group B, respectively. Cell filling in pink and gray indicates the presence or absence of a gene, respectively. Cell filling in orange indicates an amino acid mutant exiting the *pepM-*encoding protein. (**B**) Main component of the T6SS structure. An ML phylogenetic tree of VgrG1 and VgrG1 in 22 *P. antoea* strains was constructed using MEGA7 based on the alignment by ClustalW.

The comparison of genomic sequences revealed a genomic island “HIVir,” which was present in the onion pathogenic strains but absent in onion non-pathogenic strains (27). HIVir has been identified as a critical pathogenicity factor for *P. ananatis* in the production of necrotic symptoms in onions (79). Based on these findings, we analyzed 12 genes within the HIVir gene cluster across 22 *P. ananatis* strains. As shown in Fig. 7A, four strains (BD442, PA4, LCFJ-001, and S47) did not encode the HIVir gene cluster. Although 18 *P. ananatis* strains encoded the HiVir cluster, DZ-12 did not contain the *pavI* gene, and nine newly sequenced strains (JCY4, JCYB8, JCC14, JCYX9, JCC8, JCY7, CW1, JCYB5, and JCY1) lacked the *pavM* gene (Fig. 7A). In the HiVir cluster, *pepM* encodes a biosynthetic pathway for an unknown phosphonic acid natural product, which is crucial for the *hvr* operon in onion center rot (80). Sequence alignment of PepM indicated that it was conserved among the genomes of 18 *P. ananatis* strains, except for a substitution at amino acid A^249^ in B7 and M232A (Fig. S5A). This indicated that these *P. ananatis* strains could rely on a common set of HiVir clusters, particularly PepM, to induce symptoms in onions.

### T6SS in *P. ananatis* strains

Bacterial T6SS can be a nanomolecular weapon that enables bacteria to deliver effector proteins either extracellularly or directly into other bacteria or eukaryotic cells (81). The analysis of the core genes in the T6SS-1 and T6SS-2 clusters in the newly sequenced strains indicated that 21 strains encoded a complete T6SS-1 gene cluster, except for S47 (Fig. 7B and Table S7), and all strains encoded a complete T6SS-2 gene cluster, presenting that the T6SS system was conserved in most sample strains. TssI (VgrG) and Hcp proteins secreted by T6SS are essential for their assembly. Analysis of variations in VgrG across 20 *P. ananatis* strains revealed that nine strains (BD442, PA4, LMG2013, PNA97-1R, JCC8, JCYX9, CW1, JCY7, and LCFJ-001) encoded only one VgrG1 in the T6SS-1 cluster, whereas 10 strains (JCY4, JCYB8, JCC14, DZ12, PA13, 26SR6, B7, M232A, LMG2665, and JCY1) encoded both VgrG1 in the T6SS-1 cluster and VgrG2 in the T6SS-2 cluster (Fig. 6B and Table S7). Notably, the *vgrG* gene was absent in JCYB5. The findings for BD442, PA4, LMG2013, PA13, and LMG2665T were consistent with previous comparative analyses of *P. ananatis* strains (82). Based on the VgrG1 sequence, the 20 strains were categorized into four groups. Except for PA13, the VgrG2 sequences in the 10 strains exhibited high identity, indicating their conservation among *P. ananatis* strains. The Hcp protein amino sequence was identical across the 19 strains, with a 4-amino acid deletion at the start of JCY4 and a mutation of T^79^ to I in LCFJ-001 (Fig. S5B). Therefore, it is possible that different VgrG proteins encoded by each *vgrG* gene were mobilized to the T6SS baseplate under varying physiological conditions or served different roles as effectors, structural elements, or both.

## DISCUSSION

MWS has become one of the serious diseases that occur in corn production, posing a serious threat to corn yield. There are varying perspectives on the causal agent of MWS, with some studies supporting *P. ananatis*, while others point to various fungi, including *Epicoccum latusicollum* or a fungal complex involving *Phyllosticta* sp., *Phoma sorghina*, and *Sporormiella* sp.^83^–85. In this study, 10 strains of *P. ananatis* isolated from MWS-diseased maize grown in different fields in Yunnan Province were found to colonize maize leaves without causing MWS symptoms, suggesting that *P. ananatis* may not be the causal pathogen of MWS in Yunnan Province, China. Previous studies have identified diverse *P. ananatis* strains that exhibit PGP attributes and promote plant growth by improving nutrient uptake (12–14). Most *P. ananatis* isolates demonstrated the ability to produce EPS, move, fix nitrogen, and solubilize phosphate (Table 1 and Fig. S1). EPS production and cell motility are crucial for bacterial colonization and pathogenicity and can enhance the colonization of these strains under diverse environmental conditions. Nitrogen fixation and phosphate solubilization benefit plants by enriching their nutrient supplies. Notably, certain *P. ananatis* isolates (JCC14, JCY1, and JCYB8) significantly increased maize height, root length, or fresh weight, indicating their potential for biocontrol in agricultural production.

An “open” pan-genome has been observed in similar pan-genome analyses in several bacterial species. These species can inhabit a wide range of environments, exhibit diverse lifestyles, and possess efficient mechanisms for lateral gene transfer (86–88). Pan-genomic analyses of *P. ananatis* strains isolated from various hosts highlighted the challenges in assessing the genomic diversity of strains from a single geographic location or host-pathosystem (20). In this study, the genomes of *P. ananatis* isolates associated with MWS and two *P. ananatis* isolates linked to BB were sequenced and grouped into three clusters from a common ancestor. A high ANI value (>96%) was observed among the 26 selected *P. ananatis* strains, indicating substantial genetic similarity. However, lower similarity was found between these strains, especially among the four rice-isolated strains in Group C and the other 22 strains, suggesting genomic variation among the *P. ananatis* strains. This study revealed an “open” pan-genome for *P. ananatis*, implying that additional gene families may emerge as more strains are included (89). Pan-genome analysis of other *P. ananatis* strains supports this, with newly sequenced genomes identifying the strain-specific genes. This adaptability may promote *P. ananatis* colonization of diverse hosts and potentially cause diseases (20, 27).

Horizontal gene transfer (HGT), natural selection, and gene duplication are the three main drivers of adaptive evolution in microbial genomes (90). HGT typically occurs via mobile genetic elements that carry functional genes, helping some bacteria (such as acidophiles) enhance their adaptability during evolution (91). Comparative genomic analysis revealed that *P. ananatis* exhibited an “open” pan-genome with many genes related to environmental adaptation acquired at critical evolutionary nodes, particularly within Nodes 14 and 19. Although the functions of some genes in *P. ananatis* have not been experimentally characterized, their biological roles can be inferred from related studies on homologous proteins with the same structural domains in other bacteria. For example, protein glycosylation is a widely present and highly complex post-translational modification process and plays a crucial role in various biological processes (92). Additionally, LPS in the bacterial outer membrane is critical for physiology and pathogenesis and is a primary target for detection assays of Gram-negative bacteria. RadC facilitates DNA damage repair following UV and X-ray irradiation in prokaryotes (93, 94), and the *E. coli radC* gene encodes a RecG-like DNA recombination/repair function (95, 96). At Node 14, diverse genes encoding glycosyltransferases and DNA repair protein RadC showed a continuous increase, highlighting their potential significance for the establishment, survival, and virulence of *P. ananatis*. In addition, the flagellar motor can be driven by ion influx through the peptidoglycan-tethered MotA/MotB stator (97). Chemotaxis is a mechanism by which bacteria sense environmental signals through MCP and move toward favorable conditions (98, 99). T6SS contributes to bacterial abiotic stress responses, such as acid, oxidation, and osmotic stress (100), and has been reported to be involved in the regulation of virulence in *P. ananatis* on onion (24). ABC transporters play a significant role in Mn and Fe uptake and contribute to bacterial pathogenicity and virulence (101). Therefore, the acquisition of genes encoding flagellar rotary motor MotB, ABC transporters, MCPs, and T6SS-related proteins at Node19 might enhance bacterial locomotion, chemotaxis, and iron metabolism, allowing bacteria to respond better to environmental stress and gain a growth advantage under such conditions.

It has been reported that the highly virulent fish pathogen *Photobacterium damselae* loses non-essential genes related to nitrite and sulfite reduction, urea degradation, T6SS, and several toxin-antitoxin systems. The loss of urease may be crucial for the stable colonization of zooplankton vectors, paralleling the evolutionary changes observed in *Yersinia pestis*, the causative agent of human plague (102, 103). These studies suggested that the loss of some functions, which streamlines the genome, results in adaptive phenotypic diversity, which is a beneficial evolutionary process (104). The presence of phage-related sequences in bacterial genomes is a common feature of bacterial gene annotation. Previous studies have indicated that distinct epidemics and clinical presentations of the human bacterial pathogen *Streptococcus* may be partly due to the acquisition or evolution of phage-encoded virulence factors (105, 106). Seven genes encoding phage-related proteins were lost at Node 9, indicating that the loss of these phage-encoded proteins might contribute to the adaptive phenotypic diversity of *P. ananatis*. Additionally, the loss of genes encoding proteins associated with heavy metal transport (Mn^2+^ or Fe^2+^) at Node 9 suggested a putative reduced demand for these metals by *P. ananatis*.

dN/dS ratio was used to estimate non-neutral changes relative to neutral changes and indicates the degree of selective pressure on a protein-coding gene. A dN/dS ratio of less than one suggests purifying or stabilizing selection, a ratio of one indicates neutral evolution, and a ratio greater than one reflects positive or Darwinian selection. Genes under positive selection are often studied to uncover their genetic adaptations (85). Although the dN/dS ratio is not considered sensitive enough to detect gene sets under positive selection in closely related species (107), 11 positively selected genes were identified in this study. These genes are primarily involved in information storage and processing, cellular processes, and signaling. The carbon storage regulatory system (Csr) has been discovered in various bacteria, with CsrA being the central RNA-binding protein that regulates crucial processes, such as central carbon metabolism, motility, biofilm formation, and the expression of virulence factors, particularly under acid stress (108–111). In *P. ananatis* YJ76, the enhancement of glycogen accumulation by indole is positively correlated with the inhibition of *csrA* expression by indole, indicating a potential link between *csrA* regulation and indole regulatory pathways (112). The DNA-binding winged helix domain belongs to the helix-turn-helix (HTH) superfamily and plays a key role in transcription initiation complexes, binding of left-handed Z-DNA or RNA, and protein-protein interactions (113–116). Moreover, the murein-binding lipoprotein Lpp, synthesized as a precursor in the cytoplasm and transported across the cytoplasmic membrane via a protein translocation mechanism (55), has been identified to specifically contribute to the virulence of *Salmonella enterica* Serovar Typhimurium (117). OGs related to the CsrA/B/C/D system, HTH domain, and murein-binding lipoprotein Lpp were identified under positive selection, suggesting that these OGs are beneficial to the adaptability of *P. ananatis* and have been preserved by positive environmental selection.

Although T3SS can inject effector proteins to induce HR in tobacco for many Gram-negative bacteria, *P. ananatis* lacks the key pathogenic factors of T3SS (2, 20). Moreover, the HIVir operon, including pepM, is required for onion center rot and HR in tobacco (79). Analysis of HIVir sequences revealed that these genes were highly conserved among most *P. ananatis* strains. Among them, four strains (BD442, PA4, LCFJ-001, and S47) did not encode the HIVir gene cluster. S47, along with two other strains (ZFZa and GDYCa) that also lacked the HIVir gene cluster (data not shown), was unable to induce HR in tobacco and cause rot symptoms in onions, which is associated with loss of the HIVir gene cluster. Specifically, the South African isolate PA4 can cause center rot of onions, BD442 can be isolated from brown stalk rot of maize, and LCFJ-001 can be a pathogen of mulberry bacterial wilt (118, 119). The HiVir gene cluster did not play a significant role in PA4 infection in onions, whereas BD442 and LCFJ-001 lacked the ability to cause rot symptoms in onions. Therefore, the inability of S47, GDYCa, and ZFZa strains to induce HR and infect onion may be related to the expression of the HIVir gene cluster.

T6SS structural clusters have been observed in over 25% of Gram-negative bacteria, with one to six different genetic clusters present in their genomes (120). T6SS functions as an injectisome for effector proteins, including hemolysin co-regulated protein (Hcp) and valine-glycine repeat protein (VgrG) (121, 122). This system is involved in various functions, including virulence, symbiosis, biofilm formation, stress response, and interbacterial competition (100, 123, 124). Comparative genomic analyses of the *Eucalyptus* pathogenic *P. ananatis* strain LMG20103 revealed three distinct T6SS clusters (82, 125). The T6SS-1 system is essential for synthesizing a functional T6SS and contributes to virulence and bacterial competition in *P. ananatis* LMG 2665T, whereas T6SS-2 is only present in strains isolated from symptomatic plant material (24). This suggests that the T6SS of different plant pathogens was acquired from unrelated bacteria or distantly related ancestors, with T6SS-1 and T6SS-2 potentially playing key roles in the virulence of *P. ananatis* in susceptible host plants. Our results showed that 21 out of the 22 selected strains encoded a complete T6SS-1 gene cluster, with the exception of S47, whereas all 22 strains encoded a complete T6SS-2 gene cluster, indicating a high degree of conservation among *P. ananatis* strains. The findings for BD442, PA4, LMG2013, PA13, and LMG2665T are consistent with previous comparative analyses of *P. ananatis* strains (82). Few strains encoded both VgrG1 and VgrG2 in the T6SS cluster, and the *vgrG* gene was absent in JCYB5. Therefore, it is possible that different VgrG proteins encoded by each *vgrG* gene were mobilized to the T6SS baseplate under varying physiological conditions or served different roles as effectors, structural elements, or both.

## Data Availability

The raw genome sequences have been deposited at the NCBI’s Sequence Read Archive (SRA) under BioProject ID PRJNA1122924 with accession numbers SRR29778416–SRR29778427 (Table 2).

## SUPPLEMENTAL MATERIAL

Fig. S1 aem.00329-25-s0001.TIFF Evaluation of plant growth-promoting traits of 10 *P. ananatis* isolates.

Fig. S2 aem.00329-25-s0002.TIFF Anti-pathogenic activities of *P. ananatis* isolates.

Fig. S3 aem.00329-25-s0003.TIFF COG classification of unique genes.

Fig. S4 aem.00329-25-s0004.tif Pan-genome and core-genome evolution of *P. ananatis*.

Fig. S5 aem.00329-25-s0005.tif Multiple sequence alignment of PepM and VgrG1 sequences in *P. ananatis*.

Supplemental legends aem.00329-25-s0006.docx Legends for Fig. S1 to S5.

Tables S4-S7 aem.00329-25-s0007.xlsx COG categories of unique genes from *P. ananatis* strains, OGs clustered by orthoFinder, detected gene gain and loss events at key nodes, and T6SS-1-related genes and VgrG2 in 22 *P. ananatis* strains.

Table S1 aem.00329-25-s0008.xlsx COG functional assignment of genes from 12 *P. ananatis* strains.

Table S2 aem.00329-25-s0009.docx General features of *P. ananatis* genomes available on NCBI.

Table S3 aem.00329-25-s0010.xlsx ANI values.

ASM does not own the copyrights to Supplemental Material that may be linked to, or accessed through, an article. The authors have granted ASM a non-exclusive, world-wide license to publish the Supplemental Material files. Please contact the corresponding author directly for reuse.
