# Supplementary figures and images for: Comparative genomics-based insights into Pantoea ananatis strains, isolated from white spot diseased leaves of maize with plant growth-promoting attributes
Source: Appl Environ Microbiol. 2025 May 19;91(6):e00329-25. doi: 10.1128/aem.00329-25 (PMC12175523; doi:10.1128/aem.00329-25)

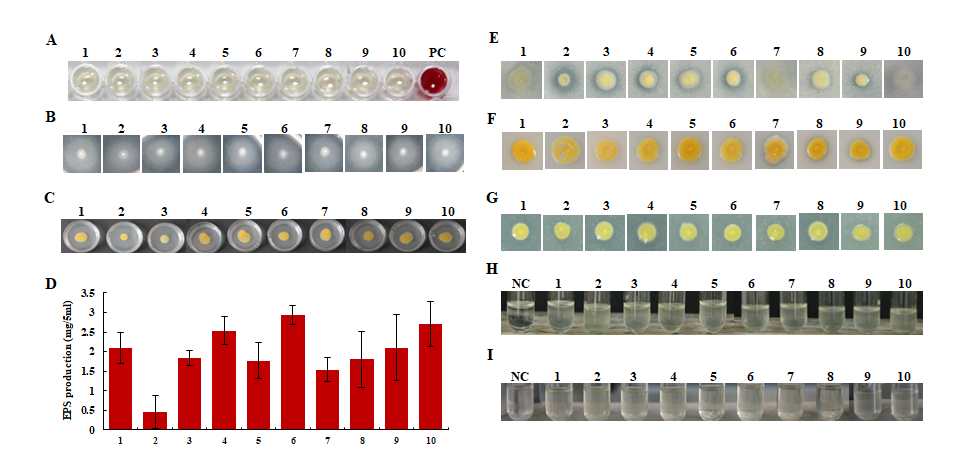

Supplement: Fig. S1 — Evaluation of plant growth-promoting traits of 10 P. ananatis isolates. [file aem.00329-25-s0001.tiff]

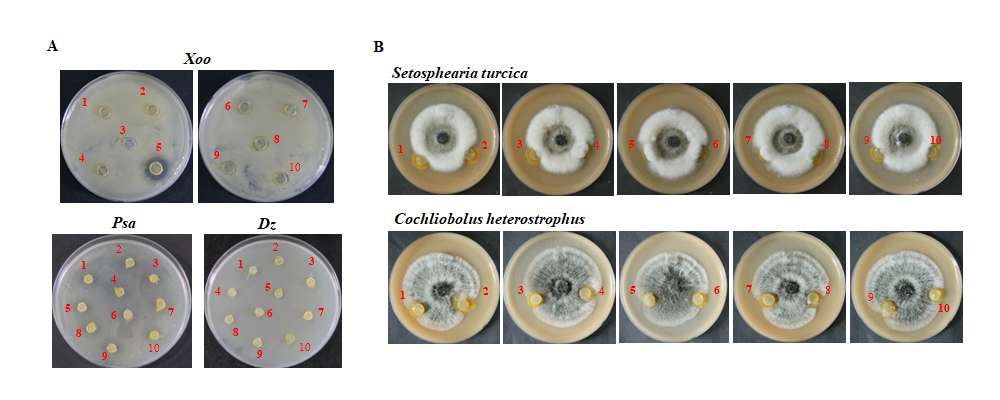

Supplement: Fig. S2 — Anti-pathogenic activities of P. ananatis isolates. [file aem.00329-25-s0002.tiff]

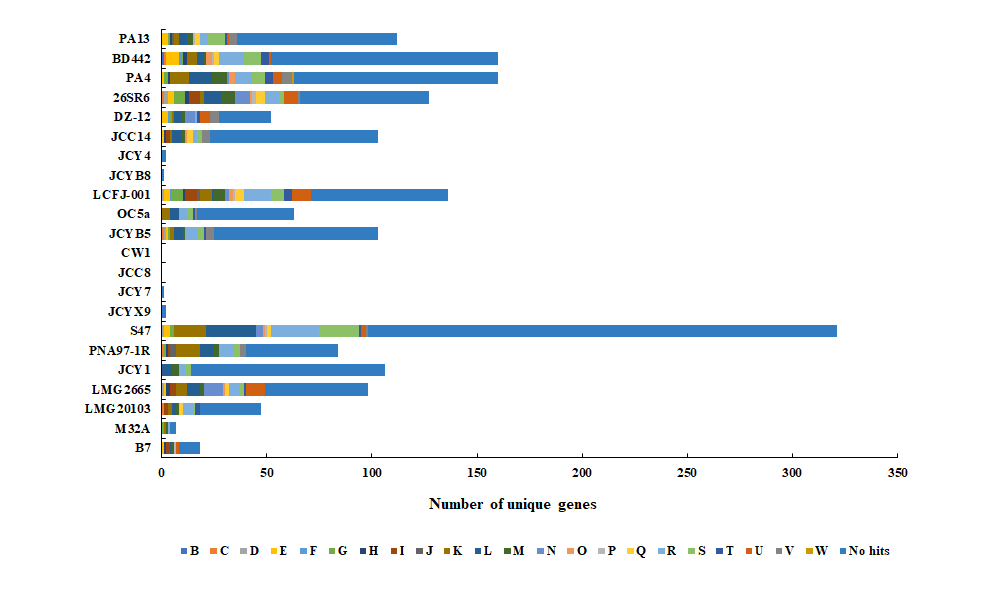

Supplement: Fig. S3 — COG classification of unique genes. [file aem.00329-25-s0003.tiff]

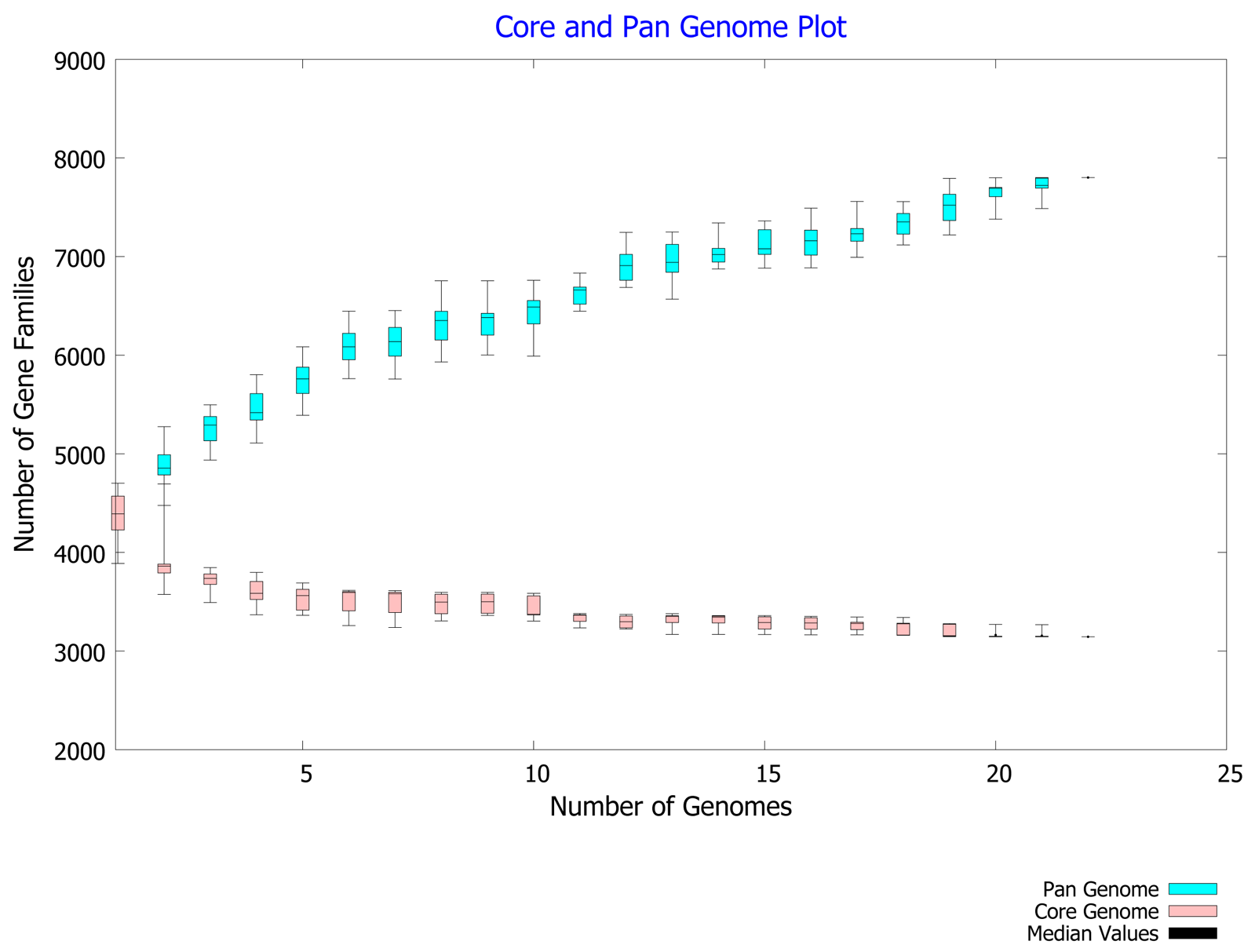

Supplement: Fig. S4 — Pan-genome and core-genome evolution of P. ananatis. [file aem.00329-25-s0004.tif]

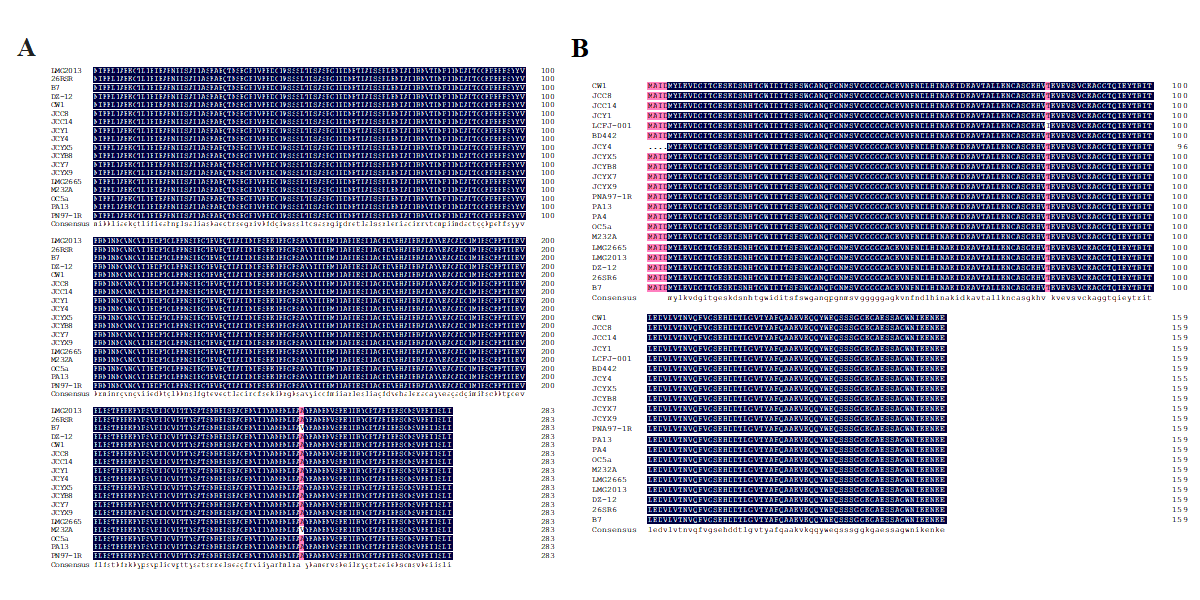

Supplement: Fig. S5 — Multiple sequence alignment of PepM and VgrG1 sequences in P. ananatis. [file aem.00329-25-s0005.tif]
