# Supplementary material for: Comparative genomics-based insights into Pantoea ananatis strains, isolated from white spot diseased leaves of maize with plant growth-promoting attributes
Source: Appl Environ Microbiol. 2025 May 19;91(6):e00329-25. doi: 10.1128/aem.00329-25 (PMC12175523; doi:10.1128/aem.00329-25)
Supplement: Supplemental legends — Legends for Fig. S1 to S5. [file aem.00329-25-s0006.docx]

**Supplemental material**

**FIG. S1 Evaluation of plant growth-promoting traits of 10 *P. ananatis* isolates.** (A) None of the *P. antoea* isolates produced IAA. All *P. ananatis* isolates displayed (B) swimming on a semi-solid plate containing 0.2% agar and (C) swarming motility on a semi-solid plate containing 0.5% agar. (D) EPS production was determined in all *P. antoea* isolates using the ethanol precipitation method. (E) Seven strains (JCYB8, S47, JCY4, CW1, JCYX9, JCY7, and JCC8) showed phosphate solubilization on the mTBA plate. Three *P. ananatis* isolates (JCYB8, JCYB5, and JCY4) exhibited proteolytic enzyme activity (F) on LB plates containing 2% skim milk. None of the *P. ananatis* isolates (G) secreted lipolytic enzymes on the mTBA plates with 1% glycerin tributyrate. All *P. ananatis* isolates showed (H) ACC deaminase activity in ADF liquid media, which contained the only nitrogen source for ACC deaminase, and (I) nitrogen fixation in nitrogen-free liquid media. Each experiment was performed in triplicates. NC, Negative control; 1, JCY1; 2, S47; 3, JCYX9; 4, JCYX7; 5, JCC8; 6, CW1; 7, JCYX5; 8, JCYB8; 9, JCY4; 10, JCC14; PC, Positive control.

**FIG. S2 Anti-pathogenic activities of *P. ananatis* isolates.** Representative results of the zone of inhibition tests for (A) bacterial pathogens and (B) fungal pathogens. JCY1 inhibits *Xanthomonas oryzae* pv. *oryzae* (*Xoo*). *Psa*, *and Pseudomonas syringae* pv. *actinidiae*; Dz, *Dickeya dadantii*; numbers1-10 indicate JCC8, S47, JCYX9, JCYX7, JCY1, CW1, JCYX5, JCYB, JCY4, and JCC14, respectively.

**FIG. S3 COG classification of unique genes.** Abbreviations: B, chromatin structure and dynamics; C, energy production and conversion; D, cell cycle control, cell division, chromosome partitioning; E, amino acid transport and metabolism; F, nucleotide transport and metabolism; G, carbohydrate transport and metabolism; H, coenzyme transport and metabolism; I, lipid transport and metabolism; J, translation, ribosomal structure, and biogenesis; K, transcription; L, replication, recombination, and repair; M, cell wall/membrane/envelope biogenesis; N, cell motility; O, post-translational modification, protein turnover, and chaperones; P, inorganic ion transport and metabolism; Q, secondary metabolite biosynthesis, transport, and catabolism; R, general function prediction only; S, function unknown; T, signal transduction mechanisms; U, intracellular trafficking, secretion, and vesicular transport; V, defense mechanisms; W, extracellular structures.

**FIG. S4 Pan-genome and core-genome evolution of *P. ananatis*.**

**FIG. S5 Multiple sequence alignment of PepM (A) and VgrG1 sequences (B) in *P. ananatis*.** Differences are marked in pink.
