# Supplementary material for: Comparative genomics-based insights into Pantoea ananatis strains, isolated from white spot diseased leaves of maize with plant growth-promoting attributes
Source: Appl Environ Microbiol. 2025 May 19;91(6):e00329-25. doi: 10.1128/aem.00329-25 (PMC12175523; doi:10.1128/aem.00329-25)
Supplement: Table S2 — General features of P. ananatis genomes available on NCBI. [file aem.00329-25-s0009.docx]

**TABLE. S2 General features of *Pantoea ananatis* genomes available on NCBI**

| **Strain** | **Source** | **Genome status** | **Accession no.** | **Completeness**  **(%)** | **Contamination (%)** | **Total bases**  **(bp)** | **GC (%)** | **Contigs** | **Coverage** | **CDSs**  **(Total)** | **Reference** |
| --- | --- | --- | --- | --- | --- | --- | --- | --- | --- | --- | --- |
| PA13 | Diseased rice grain | Complete | GCF_000233595.1 | 99.55 | 0.14 | 4,867,131 | 53.5 | 2 | 260× | 4476 | (Choi et al., 2012) |
| OC5a | Onion | Complete | GCF_017474015.1 | 99.2 | 2.52 | 4,980,968 | 53 | 4 | 118x | 4477 | (Asselin et al., 2021) |
| PNA 97-1R | Onion | Complete | GCF_002952035.2 | 99.02 | 1.77 | 4,994,140 | 53 | 3 | 200x | 4568 | (Stice et al., 2018) |
| PA4 | Onion seed | Draft | JMJK00000000  GCF_000710015.2 | 98.81 | 2.74 | 5163485 | 53.5 | 17 | 70 x | 4817 | (Weller-Stuart et al., 2014) |
| LMG 20103 | Eucalyptus | Complete | GCF_000025405.2 | 97.59 | 0.34 | 4,703,373 | 53.5 | 1 | - | 4245 | (De Maayer et al., 2010) |
| LMG 2665T | Ananas comosus fruitlets | Draft | JFZU00000000.1 | 98.76 | 1.75 | 4,980,528 | 53.5 | 28 | 380x | 4547 | (Adam et al., 2014) |
| LCFJ-001 | Mulberry | Complete | GCF_016598655.1 | 91.4 | 2.02 | 4,499,350 | 53.5 | 1 | 644x | 4061 | (Yuan et al., 2023) |
| DZ-12 | Maize | Draft | GCF_003849975.1 | 98.65 | 1.11 | 5,159,454 | 53.5 | 7 | 200x | 4663 | NA |
| BD442 | Maize stalk tissue | Draft | JMJL00000000  GCF_000709995.2 | 98.89 | 0.96 | 4,798,552 | 53.5 | 11 | 70 x | 4431 | (Weller-Stuart et al., 2014) |
| 26SR6 | Maize leaf | Draft | GCF_028555205.1 | 98.63 | 1.93 | 5,002,097 | 53 | 53 | 86.7x | 4632 | NA |
| B7 | Maize | Draft | GCF_013186345.1 | 96.19 | 3.37 | 5,168,502 | 53.5 | 95 | 85x | 4826 | NA |
| M232A | Maize leaf washing | Draft | GCF_013186335.1 | 96.23 | 3.47 | 5,167,735 | 53.5 | 89 | 73x | 4817 | NA |
| TZ39 | Rice leaf | Complete | GCF_019720835.1 | 94.99 | 3.32 | 4,895,690 | 53.5 | 1 | 100 x | 4435 | (Yu et al., 2022) |
| LT2-192 | Rice | Draft | GCF_025881975.1 | 94.19 | 3.8 | 4,965,564 | 53.5 | 69 | 150 x | 4614 | NA |

The *P. ananatis* strains identified as pathogens are marked in red.
